# Melt-electrowriting-enabled anisotropic scaffolds loaded with valve interstitial cells for heart valve tissue Engineering

**DOI:** 10.1186/s12951-024-02656-5

**Published:** 2024-06-28

**Authors:** Chao Xu, Kun Yang, Yin Xu, Xiangfu Meng, Ying Zhou, Yanping Xu, Xueyao Li, Weihua Qiao, Jiawei Shi, Donghui Zhang, Jianglin Wang, Weilin Xu, Hongjun Yang, Zhiqiang Luo, Nianguo Dong

**Affiliations:** 1 College of Materials Science and Engineering, State Key Laboratory of New Textile Materials and Advanced Processing Technology, Wuhan Textile University, No.1 Sunshine Avenue, Jiangxia District, Wuhan, 430200 China; 2 College of Life Science and Technology, Huazhong University of Science and Technology, 1037 Luoyu Road, Hongshan District, Wuhan, 430074 China; 3 Department of Cardiovascular Surgery, Union Hospital, Tongji Medical College, Huazhong University of Science and Technology, 1277 Jiefang Avenue, Wuhan, 430000 China; 4 State Key Laboratory of Biocatalysis and Enzyme Engineering, School of Life Science, Hubei University, 368 Youyi Avenue, Wuchang District, Wuhan, 430062 China

**Keywords:** Heart valve tissue engineering, Melt electrowriting, Bioactive hydrogel, Extracellular matrix remodeling, Anisotropic scaffolds

## Abstract

Tissue engineered heart valves (TEHVs) demonstrates the potential for tissue growth and remodel, offering particular benefit for pediatric patients. A significant challenge in designing functional TEHV lies in replicating the anisotropic mechanical properties of native valve leaflets. To establish a biomimetic TEHV model, we employed melt-electrowriting (MEW) technology to fabricate an anisotropic PCL scaffold. By integrating the anisotropic MEW-PCL scaffold with bioactive hydrogels (GelMA/ChsMA), we successfully crafted an elastic scaffold with tunable mechanical properties closely mirroring the structure and mechanical characteristics of natural heart valves. This scaffold not only supports the growth of valvular interstitial cells (VICs) within a 3D culture but also fosters the remodeling of extracellular matrix of VICs. The in vitro experiments demonstrated that the introduction of ChsMA improved the hemocompatibility and endothelialization of TEHV scaffold. The in vivo experiments revealed that, compared to their non-hydrogel counterparts, the PCL-GelMA/ChsMA scaffold, when implanted into SD rats, significantly suppressed immune reactions and calcification. In comparison with the PCL scaffold, the PCL-GelMA/ChsMA scaffold exhibited higher bioactivity and superior biocompatibility. The amalgamation of MEW technology and biomimetic design approaches provides a new paradigm for manufacturing scaffolds with highly controllable microstructures, biocompatibility, and anisotropic mechanical properties required for the fabrication of TEHVs.

**Supplementary Information:**

The online version contains supplementary material available at 10.1186/s12951-024-02656-5.

## Introduction

Heart valves, crucial soft tissues ensuring the correct direction of blood flow within the cardiovascular system, undergo approximately 100,000 cycles of opening and closing each day [1, 2]. Valvular heart disease impacts over 100 million individuals globally, resulting in substantial morbidity and mortality [3]. Currently employed mechanical or biological valves in clinical settings play a vital role in saving the lives of patients with severe heart valve damage or disease [4]. Nonetheless, these valve substitutes still exhibit significant drawbacks, including pronounced thromboembolic complications, extensive calcification, and a decline in durability [5, 6]. Furthermore, they lack the ability to support tissue regeneration and remodeling, a critical consideration for pediatric patients [7, 8]. 

Heart valve tissue engineering (HVTE) seeks to overcome the drawbacks of existing artificial heart valves by creating an alternative valve that offers mechanical stability, promotes tissue proliferation, and permits remodeling [9, 10]. Natural leaflets undergo around 40 million cycles of opening and closing each year, exposing them and their underlying structures to recurrent mechanical pressures [11]. The mechanical response of aortic valve leaflets demonstrates non-linear attributes, illustrating stress-strain profiles and manifesting characteristic anisotropic features of pliable biological tissues [12]. The complex reaction arises from the distinct configuration of tissue elements, including collagen and elastic fibers, predominantly clustered within the fibrous and ventricular layers of this multifaceted tissue [13, 14]. Various manufacturing techniques are utilized in crafting scaffolds for HVTE, including electrospinning, [15, 16] weaving, [17, 18] bioprinting, [19, 20] and molding, [21] with each method bearing its unique advantages. Within the realm of biofabrication technologies, solution electrospinning has attracted considerable attention for its capacity to generate ultrafine fibers that closely resemble collagen strands [22, 23]. Nevertheless, a notable disadvantage of fibrous scaffolds produced through electrospinning lies in their constrained ability to facilitate cell penetration and the absence of precise control over microarchitecture, attributed to their dense fibrous composition, resulting in inadequate porosity. Recent in vivo examinations of tissue-engineered valves have underscored this as a noteworthy issue, [24] exposing unintended accumulation of ECM predominantly on the external facets. This leads to thickening and constriction of the valve, with the potential consequence of jeopardizing cell-fiber interactions [25]. Furthermore, the capacity to spatially modify fiber structure through electrospinning is constrained [26], or on the iterative repetition of the spinning process with varied rotational axes. [27]

Melt-electrowriting (MEW) distinguishes itself as a precision-oriented biofabrication technique enabling the digital generation and additive manufacturing of fibrous scaffolds. MEW presents distinct benefits in comparison to alternative methods for fiber formation, such as traditional electrospinning. It enables accurate placement of fibers at the micrometer scale. This capacity simplifies the fine-tuning of mechanical characteristics, macro-porosities, and designs, rendering it applicable for diverse purposes, including the field of tissue engineering [28, 29] and disease simulation [30]. However, the application of MEW to engineer complex tissues, like native heart valves with distinct regions featuring specific structural properties, necessitates the integration of spatial complexity into the design. Additionally, pure 3D scaffolds exhibit drawbacks, including low hemocompatibility, limited biological activity, and insufficient regeneration capability, constraining their practical utility in HVTE.

Hydrogel emerges as one of the most promising scaffolds for soft tissue engineering. Boasting a substantial water content and molecular resemblance to natural soft tissues, hydrogels establish a dynamic and accommodating microenvironment. This environment facilitates the emulation of native extracellular matrix functions, encompassing anchorage, signaling, nutrient transport, structural integrity, and homeostasis [31]. Hydrogels possess the ability to emulate essential elements of the ECM microenvironment, thereby encouraging proper function of valve interstitial cell (VICs) and facilitating the remodeling process in engineered valve constructs [32]. Collagen serves as a pivotal structural component in all heart valves, [33] yet its sourcing is restricted. Gelatin, a hydrolyzed derivative of collagen, has attracted considerable attention due to its wide-ranging sources and biodegradable properties. With more extensive raw material source and relatively lower production costs, gelatin exhibits potential advantages. Moreover, it possesses the capability to mimic the characteristics of the ECM found in natural tissues. Specifically, Gelatin modified with methacrylate (GelMA), when exposed to UV light, is extensively employed in the fabrication of hydrogels, establishing itself as a prominent material in tissue engineering applications. Furthermore, glycosaminoglycans (GAGs) play a crucial role in resisting compressive forces during the opening and closure of all four heart valves [34]. Integrating GAGs into a collagen scaffold provides an opportunity to capitalize on the biological characteristics of these polysaccharides, thereby enhancing the functionality of HVTE scaffolds [35]. However, the mechanical strength of pure hydrogels, when used as tissue engineering scaffolds, is insufficient.

**Fig. 1:**
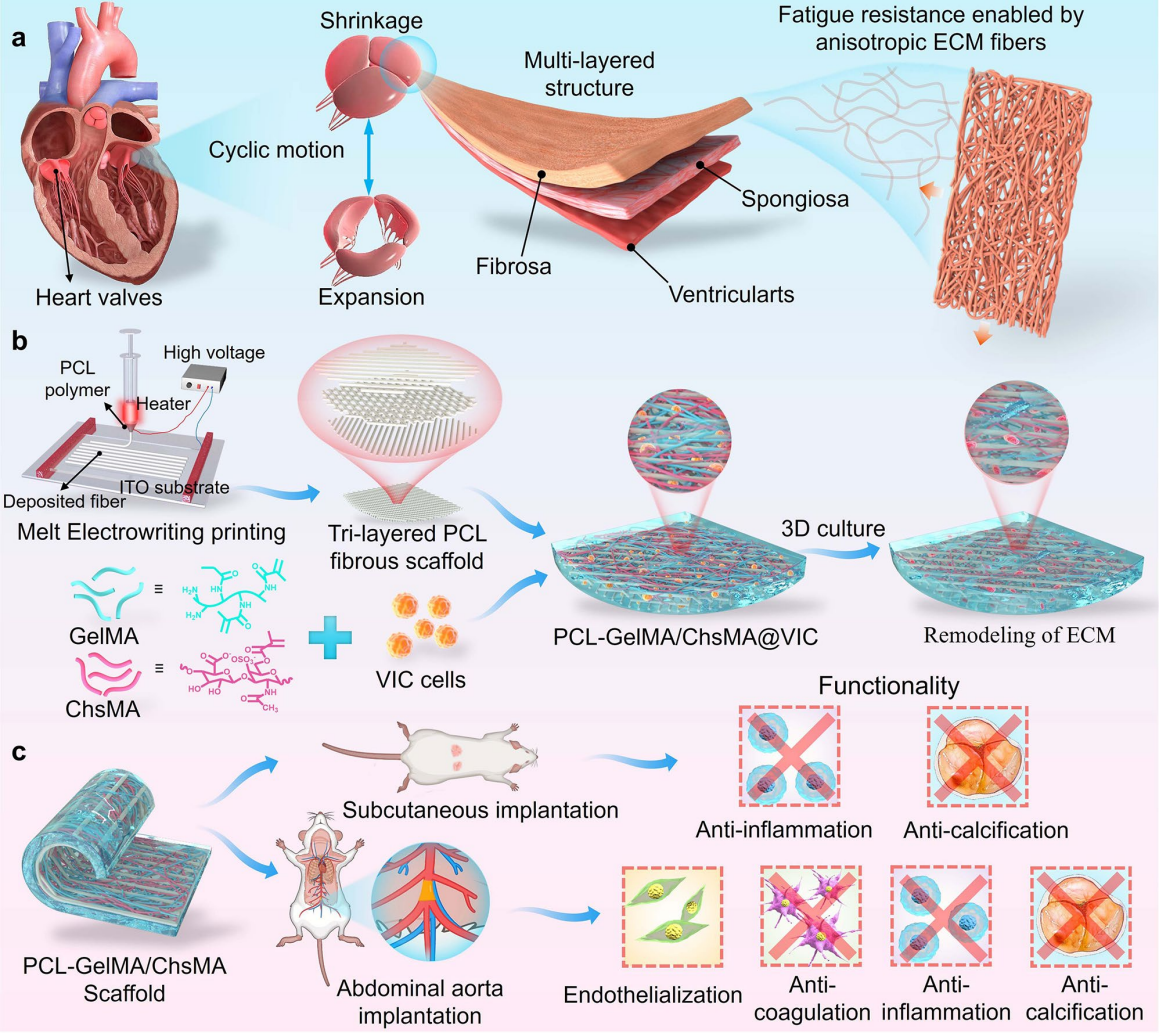
Schematic illustration of MEW-enabled biomimetic anisotropic nanofibrous scaffolds loaded with VIC for HVTE. (**a**) The heart valve has a tri-layered structure with anisotropic extra-cellular matrix to enhance its fatigue resistance. (**b**) A MEW-enabled tri-layered PCL anisotropic scaffold in combination with 3D cell culture within bioactive hydrogels (GelMA/ChsMA) was fabricated to construct a biomimetic HVTE scaffold. (**c**) In vivo functional investigation of the biomimetic HVTE scaffold

We propose that, by encapsulating VIC cells within the bioactive hydrogel and in combination with MEW scaffolds, not only a three-dimensional cell culture microenvironment is established for HVTE scaffolds, but anisotropic mechanical properties are also imparted. Herein, we present a novel MEW platform crafted to facilitate the fabrication of exceptionally versatile, structurally anisotropic, tri-layered scaffolds comprised of polycaprolactone (PCL) fibers. These scaffolds possess meticulously controlled mechanical properties, catering to the specific requirements of heart valve tissue engineering. Employing MEW techniques, we crafted tri-layered anisotropic PCL scaffolds with a biomimetically porous structure mimicking the role of collagen framework. Simultaneously, a soft gelatin methacryloyl (GelMA) hydrogel containing the bioactive polysaccharide chondroitin sulfate methacryloyl (ChsMA) was infused into the tri-layered MEW-PCL scaffold to emulate the extracellular matrix (ECM) of natural heart valves (Fig. 1). The objective of this approach was to harness the benefits inherent in both a fibrous structure and a hydrogel material, aiming to impart HVTE scaffold morphological, structural, and biomechanical heterogeneity. We illustrate that PCL-GelMA/ChsMA scaffolds effectively support the proliferation of VICs within their 3D network, which in turn facilitating the remodeling of the ECM of VICs. Through a comprehensive mechanical assessment and initial biological evaluations in both in vitro and in vivo settings, coupled with functional appraisal as an aortic valve, we systematically explore the applicability of PCL-GelMA/ChsMA scaffolds for HVTE.

## Materials and methods

### Preparation of MEW-PCL scaffolds

Scaffolds biologically inspired to replicate the anisotropic tri-layered structure were crafted utilizing a MEW device (BioMaker 4, SunP (Beijing) Biotechnology Co., Ltd.). Briefly, medical grade PCL pellets (80 kDa, aladdin) were loaded into a stainless-steel syringe, and the syringe was heated to 80 °C to get a homogeneous polymer melt. Subsequently, the melting PCL was squeezed out through a 20 G needle at a 5.5 mm s^− 1^ movement speed and 0.02 mm^3^ s^− 1^ quantity of flow, where high voltage of 3.5 kV drags the PCL fiber downward onto conductive glass collector approximately 2 mm away from the needle.

### Preparation of methacrylic anhydride modified gelatin (GelMA) and chondroitin sulfate (ChsMA)

Gelatin (porcine skin, type A), chondroitin sulfate (Chs), methacrylic anhydride (MA) and the photoinitiator lithium phenyl-2,4,6-trimethylbenzoylphosphinate (LAP) were purchased from Sigma-Alrich. The synthesis of GelMA and ChsMA involved the reaction of gelatin and Chs, respectively, with MA, following established methods. [36, 37] In brief, gelatin and Chs were dissolved in deionized water at concentrations of 10% and 20%, respectively. Gelatin underwent a one-hour reaction at 60℃ with 0.5 g of MA per gram of gelatin. Simultaneously, Chs underwent a 24-hour reaction with MA at 30℃, with pH adjustments maintained at 8 using sodium hydroxide. The molar excess of MA over the hydroxyl groups for Chs was tenfold. Following the reaction period, insoluble MA was separated through centrifugation, succeeded by dialysis against deionized water to eliminate any residual unreacted MA and methacrylic acid, followed by freeze-drying and storage at -20℃.

The MEW-PCL scaffold was integrated into GelMA/ChsMA hydrogel by suspending VIC cells (2 million cells mL^− 1^) in the polymerization starting solution (10 wt% GelMA, 0.5 wt% ChsMA, and 0.05 wt% LAP). The MEW-PCL scaffold was filled with the components of the GelMA/ChsMA hydrogel. The rapid polymerization of the GelMA/ChsMA prepolymer solution occurred under UV light exposure (10 mW cm^− 2^, 365 nm) for 15 s to achieve gelation, ensuring a consistent distribution of cells throughout the graft.

### Characterization of MEW-PCL scaffolds and hydrogel-embedded PCL scaffolds

The morphological evaluations of all the scaffolds, comprising MEW-PCL scaffolds, PCL-GelMA scaffolds, and PCL-GelMA/ChsMA scaffolds, were conducted using a scanning electron microscope (SEM, FEI, The Netherlands). The rheological behavior of GelMA, GelMA/ChsMA hydrogels, and hydrogel-embedded PCL scaffolds (PCL-GelMA and PCL-GelMA/ChsMA) was assessed at room temperature with a rheometer (Kinexus, Malvern). The storage modulus (G’) and loss modulus (G’’) of the hydrogels were examined in the dynamic frequency range of 0.1 to 10 Hz at a 0.5% strain amplitude.

A tensile strength tester (CMT4503, Metz Test Technology Co., Ltd) was utilized to assess the mechanical properties of various scaffold samples under wet conditions. Rectangular samples (30 mm × 10 mm) were prepared and for testing. Tensile experiments were conducted with a standardized gauge length of 10 mm and a constant displacement rate of 10 mm min^− 1^. The typical stress-strain curves were analyzed to determine the Young’s modulus, yield strength, and yield elongation of the samples.

The water contact angle was measured using a contact angle system. Microdroplets of liquid were dispensed onto the surface via a micro syringe, and images were captured using a camera for subsequent calculation of the water contact angle.

### Endothelialization

The specimens of PCL, PCL-GelMA, and PCL-GelMA/ChsMA scaffolds (with a diameter of ϕ = 8 mm and a sample size of *n* = 6) underwent sterilization in 75% ethanol for 24 h, followed by immersion in PBS to eliminate residual ethanol. A total of 2 × 10^5^ HUVECs were seeded onto the scaffolds and cultured at 37 °C for 1 and 3 days, respectively. The quantification of HUVECs on the scaffolds was conducted by CCK-8 assay. Additionally, a live/dead staining assay was carried out by employing Calcein-AM and PI, while cell morphology was visualized through staining with DAPI and TRITC-phalloidin. The observations were made by a fluorescence microscope.

The cell migration assay was conducted following a previously described protocol [38]. Briefly, PCL-GelMA and PCL-GelMA/ChsMA specimens (with a diameter of ϕ = 8 mm) were subjected to sterilization in 75% ethanol for 24 h. Subsequently, 2 × 10^5^ HUVECs were seeded onto the specimens. After an incubation period at 37 °C for 24 h, a confluent monolayer of cells was created, and a scratch was made using a 10 µL pipette tip. The scratched area was then washed with PBS, and HUVECs were cultured for an additional 0, 24, and 48 h. The scaffolds were stained using DAPI and phalloidin, and captured by a fluorescence microscope.

### In Vitro Hemocompatibility Assay

#### Platelet adhesion

The whole rabbit blood was collected and centrifugation at 3000 rpm for 5 min to prepare platelet-rich plasma (PRP). Specimens of PCL, PCL-GelMA, and PCL-GelMA/ChsMA were then incubated with 100 µl of PRP at 37 °C for 1 h. Subsequently, the PRP was removed, and the specimens were thoroughly rinsed with PBS. For scanning electron microscopy characterization, the specimens were fixed with 4% paraformaldehyde for a duration of 1 h, followed by triple rinsing with PBS. The specimens underwent a dehydration process using a series of gradient alcohol solutions (60%～100%) and were imaged using a SEM (FEI, The Netherlands). The quantitative assessment of platelet adhesion involved measuring the content of LDH released from platelets using the LDH kit (Beyotime, China).

#### Hemolysis analysis

Red blood cells (RBCs) were harvested from rabbit whole blood after undergoing centrifugation at 6000 revolutions per minute for 10 min. PBS served as the medium for a tenfold dilution of the red blood cells (RBCs). Subsequently, PCL, PCL-GelMA, and PCL-GelMA/ChsMA scaffold specimens (with a diameter of ϕ = 8 mm) were dispersed into 200 µL PBS and positioned in a 48-well plate. Each well was filled with 800 µL of the red blood cell diluted solution. Positive and negative controls were established using Distilled Water (DW) and PBS, respectively. After an incubation period of 2 h at 37 °C, the supernatant was separated via centrifugation, and 100 µL of the supernatant from all specimens was transferred to a 96-well plate. The absorption at 541 nm was subsequently determined using a microplate reader (Multiskan GO, ThermoScientific, USA). The hemolysis ratio was computed using the formula: Hemolysis Ratio = Absorption (Sample) / Absorption (DW) × 100%.

### Extracellular matrix remodeling analysis

2 × 10^5^ VIC mixed with hydrogel and was injected into a PCL scaffold, cultured for a period of 14 days. Following this, the constructs were harvested and fixed in 4% paraformaldehyde. The scaffolds samples were incubated overnight at 4 °C with primary antibodies specific to vimentin (ab8978, abcam), α-SMA (ab7817, abcam), Collagen I (ab6308, abcam), Collagen III (68320-1-Ig, proteintech), and Elastin (E4013, Sigma). Following the primary antibody incubation, the samples were exposed to secondary fluorescent antibodies for 2 h and subsequently treated with DAPI nuclear dye for 10 min. Finally, the stained samples were visualized using a Confocal Laser Scanning Microscope (FV3000, Olympus).

### Subcutaneous implantation

Animal experiments adhered to the ethical standards established by the Institutional Animal Care and Use Committee of Huazhong University of Science and Technology. To assess the in vivo histocompatibility of the scaffolds, a rat subdermal implantation model was employed. Scaffold samples, sized at 1 cm × 1 cm, were sterilized with 75% ethanol. Following this, implantation procedures were carried out on male SD rats (200 ± 10 g, Hubei Laboratory Animal Research Center). Surgical incisions were made on the dorsal area of each rat using surgical scissors. Subsequently, scaffold samples were inserted into the subcutaneous pocket, and the incisions were closed using surgical staples. Detailed operational procedures can be found in the supporting information. After 30 days of implantation, the samples were removed and subjected to characterization using HE staining, macrophage markers (CD68, iNOS, CD206, and Arg-1), and alizarin red staining to demonstrate the in vivo histocompatibility of the scaffolds and assess calcification.

### In vivo rat abdominal aorta implantation model

To evaluate the scaffolds’ performance in a hemodynamic environment, we established a rat abdominal aorta implantation model. Detailed operational can be referenced in the supplementary information. Following a 4-week interval, the scaffolds underwent immunofluorescent staining involving HE, iNOS, CD206, CD31, Vimentin, and alizarin red. Macrophage markers (iNOS and CD206), endothelial cell marker (CD31), interstitial cell marker (vimentin), and alizarin red were employed to assess inflammation, cellularization, and calcification of the implanted scaffolds, respectively.

### Statistical analysis

Quantitative data are presented as mean ± standard deviation (SD). All experiments were conducted with a minimum of three replicates per group. Statistical analyses were performed using ANOVA with Scheffé post hoc test for multiple group comparisons and unpaired t-test for two-group comparisons. A significance level of *p* < 0.05 was considered statistically significant.

## Results and discussion

### Biomimetic design and fabrication of melt-electrowriting scaffolds

**Fig. 2:**
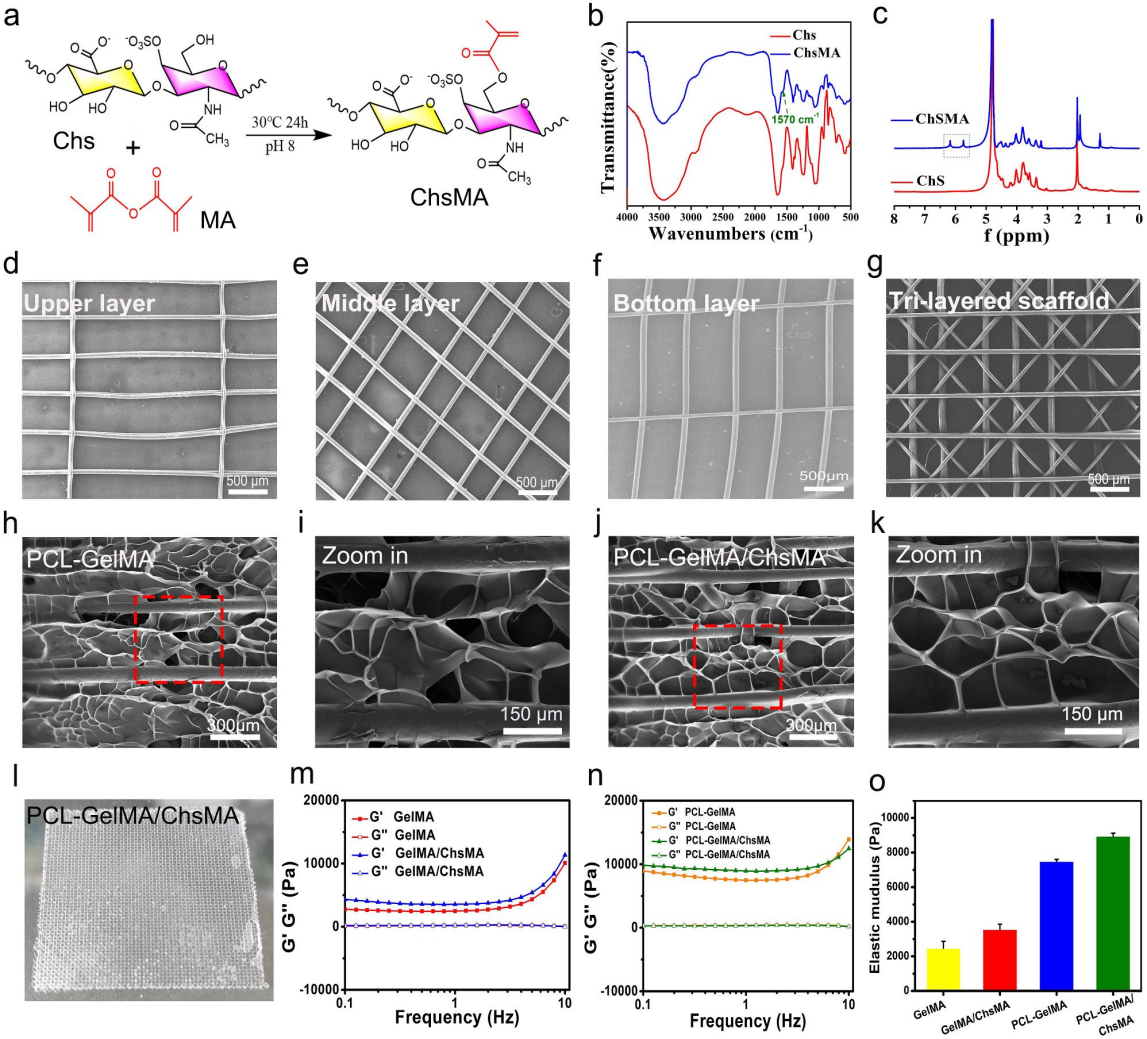
Design and fabrication of MEW-enabled biomimetic anisotropic scaffolds for HVTE. (**a**) Diagram of modification reaction of chondroitin sulfate methacrylate. (**b**) FTIR spectra show the comparison before and after the modification of chondroitin sulfate with methacrylate anhydride. (**c**) ^1^H NMR spectra of chondroitin sulfate and chondroitin sulfate methacrylamide. The SEM images display (**d**) transverse-aligned fibers on the top layer, (**e**) diamond-shaped fibers on the middle layer, and (**f**) vertically aligned fibers on the bottom layer of the tri-layered anisotropic MEW-PCL scaffolds. (**g**) SEM images of tri-layered anisotropic MEW-PCL scaffolds. SEM images of the GelMA-hydrogel-filled MEW-PCL composite scaffold (**h**) and a local magnification image (**i**). SEM images of the GelMA/ChsMA-hydrogel-filled MEW-PCL composite scaffold (**j**) and a local magnification image (**k**). (**l**) A photograph of GelMA/ChsMA-hydrogel-filled MEW-PCL composite scaffold. (**m**) G’ and G’’ of GelMA and GelMA/ChsMA scaffolds. (**n**) G’ and G’’ of PCL-GelMA and PCL-GelMA/ChsMA composite scaffolds. (**o**) The elastic modulus (G’) at 1 Hz of different scaffolds

Our study aims to create a synthetic leaflet with hydrogel-filled tri-layered PCL composite scaffold, which is meticulously designed to mirror the structural and mechanical attributes of natural heart valves. Native heart valve tissues exhibit intricate 3D structures comprising multiple interconnected layers, and the architecture of these layers plays a pivotal role in determining their mechanical characteristics and functionality [39]. The crucial mechanical properties of these tissues are anisotropic, which arises from the alignment of fibers within the extracellular matrix (ECM). The natural valve tissue primarily consists of collagen, with a small amount of polysaccharide [34]. To simulate the composition of natural valves, we initiated the process by preparing GelMA and ChsMA molecules. These molecules were synthesized by reacting gelatin and chondroitin sulfate (Chs) with methacrylic anhydride, as illustrated in Fig. 2a. Figure S1 illustrates the FTIR and 1H NMR spectra of gelatin and GelMA, revealing a novel peak at 1564 cm^− 1^ in the FTIR spectrum of GelMA and the emergence of two additional peaks at 5.6 and 5.4 ppm in the 1H NMR spectrum. These findings affirm the successful grafting of methacrylic anhydride onto gelatin. ChsMA was also successfully obtained, as confirmed by FTIR and ^1^H NMR spectra. The stretching band of the C = C bond from methacrylate at 1570 cm^− 1^ was detected in ChsMA (Fig. 2b). The presence of two distinct peaks at 5.6 and 6.1 ppm was ascribed to the two protons linked to the CH = CH_2_ bond of methacrylate (Fig. 2c). We then employed a Bio-3D printer equipped with an electrostatic direct writing nozzle to fabricate various poly(ε-caprolactone) (PCL) MEW scaffolds. Initially, we designed and printed monophasic scaffolds, which included transverse-aligned fibers, diamond-shaped fibers, and vertically-aligned fibers architectures (Fig. 2d-f). Subsequently, we proceeded to print tri-layered anisotropic PCL MEW scaffolds in one package (Fig. 2g). The mean fiber diameter within the constructs was determined to be 55 ± 1.5 μm. Notably, this dimension is smaller than the typical fiber diameters of the 3D-printed scaffolds manufactured using conventional melt extrusion techniques such as fused deposition modeling and extrusion. Typically, these conventional methods result in fiber diameters exceeding 200 μm [40]. 

The MEW-PCL scaffolds were integrated into hydrogel via molding, generating hybrid constructs that harness the strengths of each constituent. Precisely, these hybrid constructs derive advantages from the customized mechanical properties and biomimetic microarchitecture provided by the fiber phase. The validation of these structural characteristics was accomplished through a morphological examination of SEM images (Fig. 2h-k). The GelMA and GelMA/ChsMA hydrogel, when introduced into the PCL MEW scaffold, forms a tightly bound dimensional pore structure. The actual appearance of this composite scaffold is depicted in Fig. 2l, revealing a tough, multilayered structure. A deeper understanding of the viscoelastic properties of these composite scaffolds was obtained through a frequency sweep analysis covering a wide range of frequencies (0.1–10 Hz). As illustrated in Fig. 2m-o, both GelMA and GelMA/ChsMA hydrogel exhibited similar nonlinear rheological behaviors, with an elastic modulus of about 2000 Pa. It’s noteworthy that the storage modulus (G’) and loss modulus (G’’) noticeably increased when MEW-PCL scaffold was embedded in the hydrogel.

### Characterization of bioactive-hydrogel-embedded composite scaffold

**Fig. 3:**
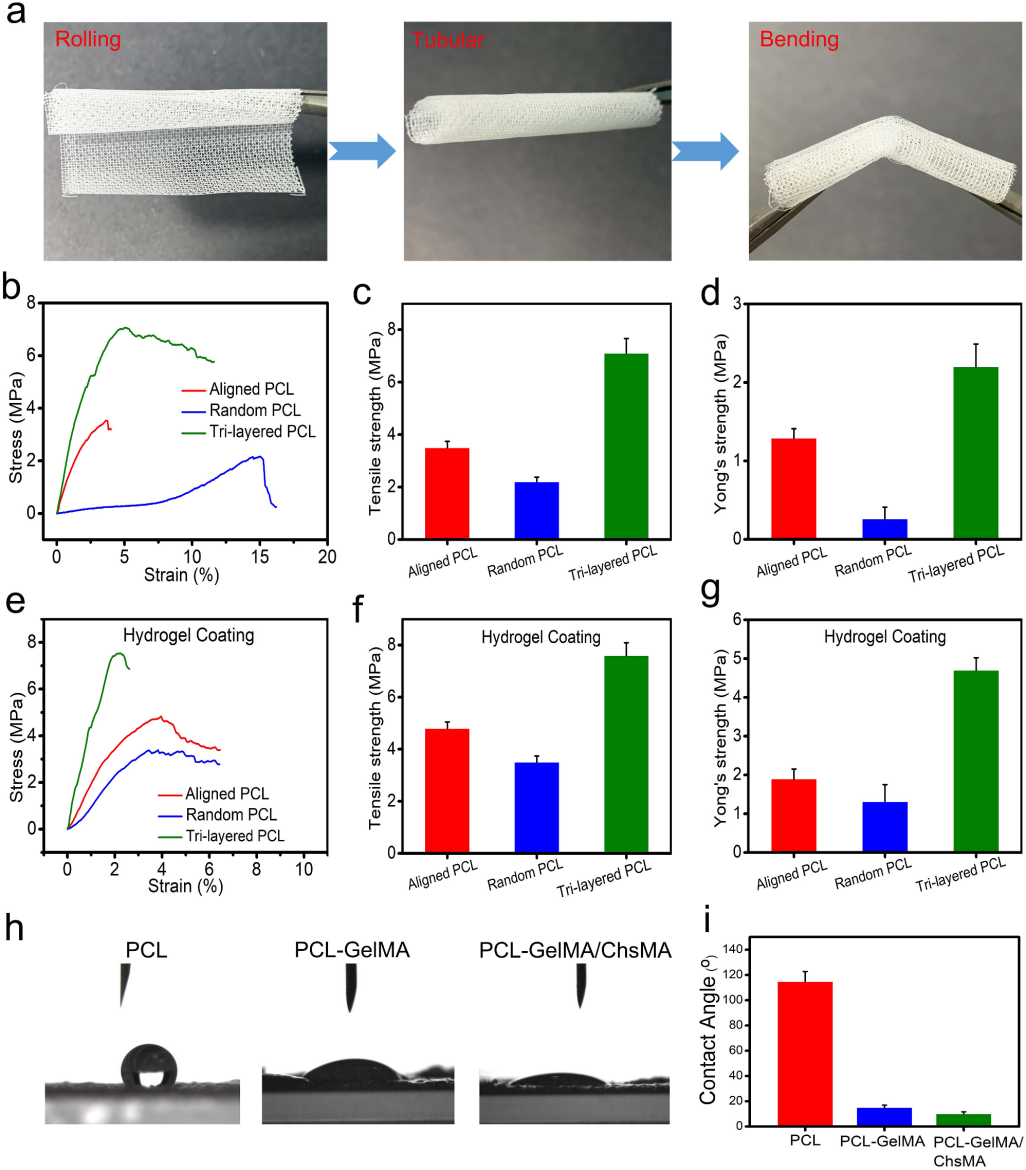
Mechanical and hydrophilic properties of tri-layered anisotropic MEW-PCL scaffolds and the hydrogel-filled composite scaffolds. (**a**) Photographs of rolled, tubular, and bent tri-layered anisotropic MEW-PCL scaffolds. (**b**) Representative stress-strain curves of each monophasic scaffold (aligned PCL fibers and random PCL fibers) and tri-layered anisotropic MEW-PCL scaffolds. (**c**) Young’s modulus of each monophasic scaffold and tri-layered anisotropic MEW-PCL scaffolds. (**d**) Ultimate tensile strength of each monophasic scaffold and tri-layered anisotropic MEW-PCL scaffolds. (**e**) Representative stress-strain curves of GelMA/ChsMA-hydrogel-filled tri-layered PCL composite scaffold. (**f**) Young’s modulus of GelMA/ChsMA-hydrogel-filled tri-layered PCL composite scaffold. (**g**) Ultimate tensile strength of GelMA/ChsMA-hydrogel-filled tri-layered PCL composite scaffold. (**h**) Images of water droplet contacting different scaffolds. (**i**) The water contact angle of different scaffolds

We conducted uniaxial tensile experiments to evaluate the influence of a precisely regulated anisotropic structure on the mechanical characteristics. Uniaxial testing has been extensively utilized in characterizing the mechanical behavior of native valvular tissue, [41] providing benchmark values essential for the advancement of scaffolds in HVTE. Before tensile testing, we do a crimp test on the scaffold to see how strong it is. The result is shown in Fig. 3a. MEW-PCL scaffold has good toughness, so that it can be rolled into a tube shape and bent. To showcase the viability of employing PCL-GelMA/ChsMA for the heart valves, a 3D-printed valve stent was manufactured. The valve stent, depicted in Figure S2a, possessed a diameter of 20 mm. The PCL-GelMA/ChsMA leaflet was positioned circumferentially and successfully enveloped around the valve stent with the use of cyanoacrylate. The leaflet placement resulted in an excess length of 5 mm above the valve post, as measured. In Figure S2b-d, the mounted PCL-GelMA/ChsMA leaflet is observed in open, semi-closed, and tightly closed states, demonstrating its potential for advancement as scaffolding for heart valves.

Figure 3b shows the tensile properties of the tri-layered MEW-PCL scaffold and its individual layers. The tri-layered MEW-PCL scaffold displayed a markedly higher tensile strength, registering at 7.1 ± 0.56MP, compared to the aligned PCL fibers (3.5 ± 0.24 MPa) and random PCL fibers (2.2 ± 0.18 MPa) (Fig. 3c). The Young’s modulus of the MEW-PCL scaffold was (2.2 ± 0.29 MPa), which was notably higher for the aligned PCL fibers (1.29 ± 0.12 MPa) and random PCL fibers (0.26 ± 0.15 MPa) (Fig. 3d). The mechanical properties of GelMA/ChsMA-bioactive-hydrogel-embedded composite MEW-PCL scaffold have improved slightly (Fig. 3e). Ultimate tensile strength of GelMA/ChsMA-hydrogel-embedded aligned PCL fibers and random PCL fibers was 4.8 ± 0.25 MPa and 3.5 ± 0.23 MPa, respectively, while ultimate tensile strength of GelMA/ChsMA-hydrogel-embedded tri-layered MEW-PCL scaffold was 7.6 ± 0.49 MPa (Fig. 3f). The Young’s modulus of the MEW-PCL scaffold also increased with the embedment of the hydrogel. The Young’s modulus of GelMA/ChsMA hydrogel-embedded aligned PCL fibers and random PCL fibers was 1.9 ± 0.24 MPa and 1.3 ± 0.42 MPa, while GelMA/ChsMA hydrogel-embedded tri-layered MEW-PCL scaffold was 4.7 ± 0.32 MPa (Fig. 3g). We further evaluated the hydrophilicity of specimens by the water contact angle (Fig. 3h). The contact angles of MEW-PCL scaffold, PCL-GelMA scaffold, and PCL-GelMA/ChsMA were 114.7°, 15.3°, and 10.6°, respectively, which indicated that hydrogels can significantly improve the hydrophilicity of the tri-layered MEW-PCL scaffold.

### In vitro cytotoxicity and endothelial cell growth and proliferation

**Fig. 4:**
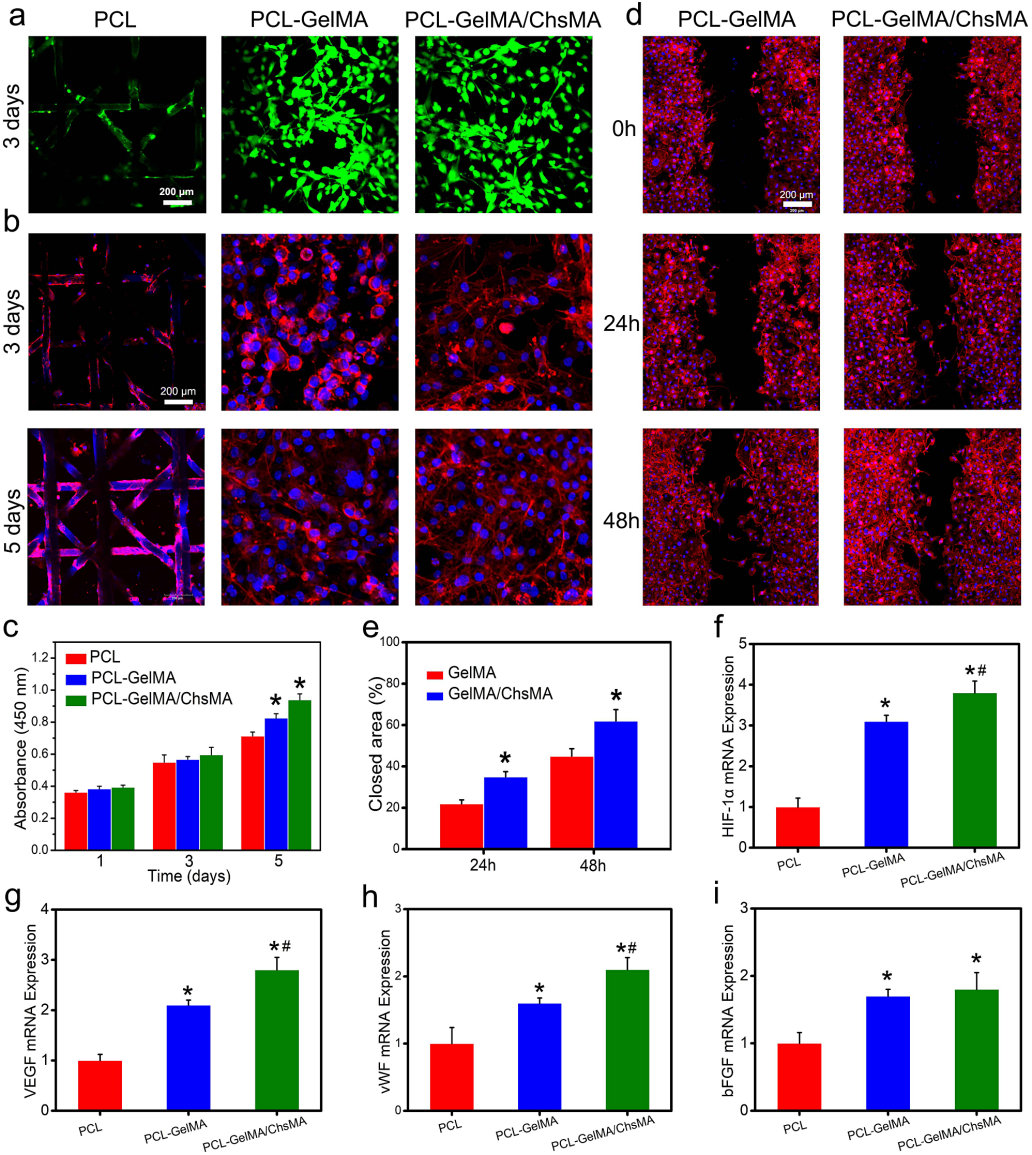
Biological behaviors of HUVECs on the HVTE scaffolds. (**a**) Live and dead staining of HUVECs grown on the surface of MEW-PCL scaffold and hydrogel-filled MEW-PCL scaffold after incubation for 3 days. (**b**) F-actin and DAPI staining of HUVECs grown on the surface of scaffolds after 3 and 5 days of incubation. (**c**) Cell viability of HUVECs on the surface of scaffolds after 1, 3 and 5 days of incubation. (**d**) Representative images of scratch wound healing assay at 0, 24, and 48 h. (**e**) Percentage of wound closure determined by the microscopic photographs. (f-i) The mRNA expression level of HIF-1α, VEGF, vWF and bFGF in HUVECs after culturing with different scaffolds for 7 days. The presented data are expressed as means ± SD (*n* = 6). A significance level of *p* < 0.05* denotes a significant difference compared to the PCL group, while *p* < 0.05^#^ indicates a significant difference in comparison to the PCL-GelMA group

The integration of endothelial cells in bioprosthetic heart valves holds significant importance for anti-coagulant and anti-calcification functionalities [42]. To assess the potential for endothelialization, we utilized the HUVEC viability assay to assess the specimens [43]. Live and dead staining of HUVECs grown on the surface of scaffolds reveals a significant presence of viable cells on both the MEW-PCL scaffold and hydrogel-filled PCL scaffolds. This observation suggests the exceptional biocompatibility of both the MEW-PCL scaffold and the hydrogel-filled PCL scaffolds, as illustrated in Fig. 4a. Observations of HUVEC morphologies on the scaffolds were conducted through actin staining. Figure 4b indicates that HUVECs exhibited excellent spreading and adhesion on the hydrogel-filled PCL scaffolds. Conversely, the MEW-PCL scaffold displayed larger apertures, proving challenging to support cell adhesion and growth. Additionally, HUVECs assumed their typical elongated shape on the hydrogel-filled PCL scaffolds. Furthermore, cellular viability of HUVECs on the specimens was evaluated using the CCK-8 assay (Fig. 4c). HUVECs cultured on hydrogel-filled PCL scaffolds displayed continuous proliferation over a 5-day incubation period, exhibiting higher absorbance values compared to those on the MEW-PCL scaffold. These results suggest that the hydrogel-filled PCL scaffolds enhanced the proliferation of HUVECs. Altogether, these findings highlight the GelMA/ChsMA hydrogel’s capability in providing a biocompatible microenvironment conducive to HUVEC growth.

Next, cell migration test and RT-qPCR analysis were used to analyze the endothelialization of HUVECs on hydrogel-embedded scaffolds. Cellular migration plays a crucial role in wound healing, particularly for HUVECs, as it accelerates the endothelialization process [44]. In Fig. 4d, it is evident that cell migration was notably enhanced in the PCL-GelMA/ChsMA group when compared to the PCL-GelMA group. At the 48-hour mark, the PCL-GelMA/ChsMA group exhibited a significantly improved closure effect. Quantitative analysis further indicated that the PCL-GelMA/ChsMA group demonstrated a superior closure effect compared to the PCL-GelMA group (Fig. 4e), which should be mostly attributable to bioactive chondroitin sulfate. Endothelial growth factors are important in the modulation of endothelial cell behaviors. Therefore, we further investigation the expression of endothelial growth factor-related genes in HUVECs. After culturing HUVECs on hydrogel-embedded PCL scaffolds for 7 days, we evaluated the gene expression levels of HIF-1α, VEGF, vWF, and bFGF. The gene expression analysis of HUVECs cultivated on PCL-GelMA/ChsMA reveals an elevated level of gene expression, specifically for HIF-1α, VEGF, vWF, and bFGF, as illustrated in Fig. 4f-i. These findings indicate that the PCL-GelMA/ChsMA scaffold exhibits favorable biocompatibility, and supports the growth and proliferation of endothelial cells. Consequently, these results position the PCL-GelMA/ChsMA scaffold as a promising candidate for the application of TEHVs.

### In vitro biocompatibility and hemocompatibility

**Fig. 5:**
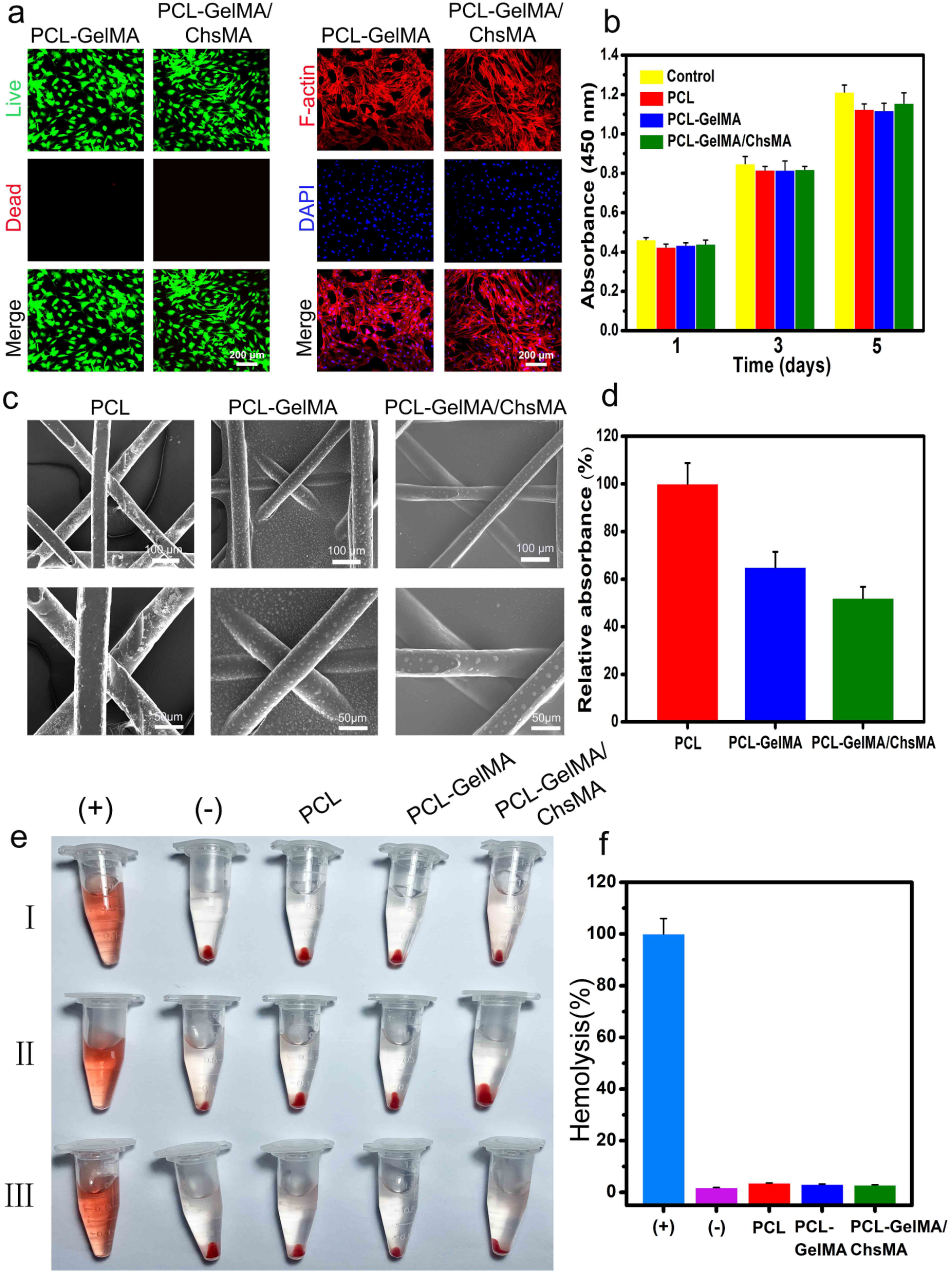
Biocompatibility and hemocompatibility of the HVTE scaffolds. (**a**) Live & dead staining and F-actin staining of VICs seeded on PCL-GelMA and PCL-GelMA/ChsMA scaffolds after a 5-day incubation period. (**b**) Cell viability of VICs on the surface of scaffolds after incubation for 1, 3 and 5 days. (**c**) SEM images of PCL, PCL-GelMA and PCL-GelMA/ChsMA scaffolds after platelet adhesion assay. (**d**) Quantitative results of platelets adsorbed on PCL, PCL-GelMA, and PCL-GelMA/ChsMA scaffolds, which was determined by the LDH release assay. (**e**) Hemolysis results of different scaffolds (“+” indicating the positive control group and “-” indicating the negative control group). (**f**) The hemolysis ratio of different scaffolds

Hemocompatibility is a critical assessment parameter for materials intended for blood contact [45, 46]. Before delving into the investigation of the hemocompatibility, the biocompatibility of MEW-PCL scaffolds to VICs was examined. As depicted in Fig. 5a, live/dead-stained images of VICs on PCL-GelMA and PCL-GelMA/ChsMA scaffolds reveal a significant presence of living cells on both scaffolds, indicating excellent biocompatibility for both PCL-GelMA and PCL-GelMA/ChsMA scaffolds. The morphologies of VICs on the scaffolds were further examined through actin staining. As depicted in Fig. 5a, the VICs exhibited good spreading and attachment on the scaffolds. Moreover, the biocompatibility of the hydrogels was assessed using the CCK-8 assay (Fig. 5b). VICs cultured on the scaffolds displayed continuous proliferation over a 5-day incubation period, with no significant difference observed compared to the control group (culture plate). This suggests that both the PCL scaffold and hydrogel-filled PCL scaffolds were not cytotoxic to VICs. These findings underscore that the PCL scaffold and hydrogel-filled PCL scaffolds provide a biocompatible microenvironment conducive to the growth of VICs.

To investigate the hemocompatibility of the PCL-GelMA and PCL-GelMA/ChsMA scaffolds, SEM was employed to examine platelet adhesion on these scaffolds. In Fig. 5c, numerous platelets adhered to the surface of the PCL scaffold, whereas only a few were observed on the surfaces of PCL-GelMA and PCL-GelMA/ChsMA scaffolds. The lactate dehydrogenase (LDH) release assay further demonstrated that the platelet adhesion on PCL-GelMA and PCL-GelMA/ChsMA scaffolds was significantly lower than that on the PCL scaffold (Fig. 5d). Additionally, the hemolysis test results shown in Fig. 5e indicated no apparent hemolysis phenomenon in the PCL, PCL-GelMA, and PCL-GelMA/ChsMA scaffold groups. The hemolysis ratios for PCL, PCL-GelMA, and PCL-GelMA/ChsMA scaffolds were 3.6%, 3.1%, and 2.8%, respectively (Fig. 5f). These hemolysis rates were well below 5%, meeting the requirements of ISO 5840-3.2013 [47]. This underscores the satisfactory hemocompatibility of the PCL, PCL-GelMA, and PCL-GelMA/ChsMA scaffolds.

### In vitro biological activity evaluation of the HVTE scaffold

**Fig. 6:**
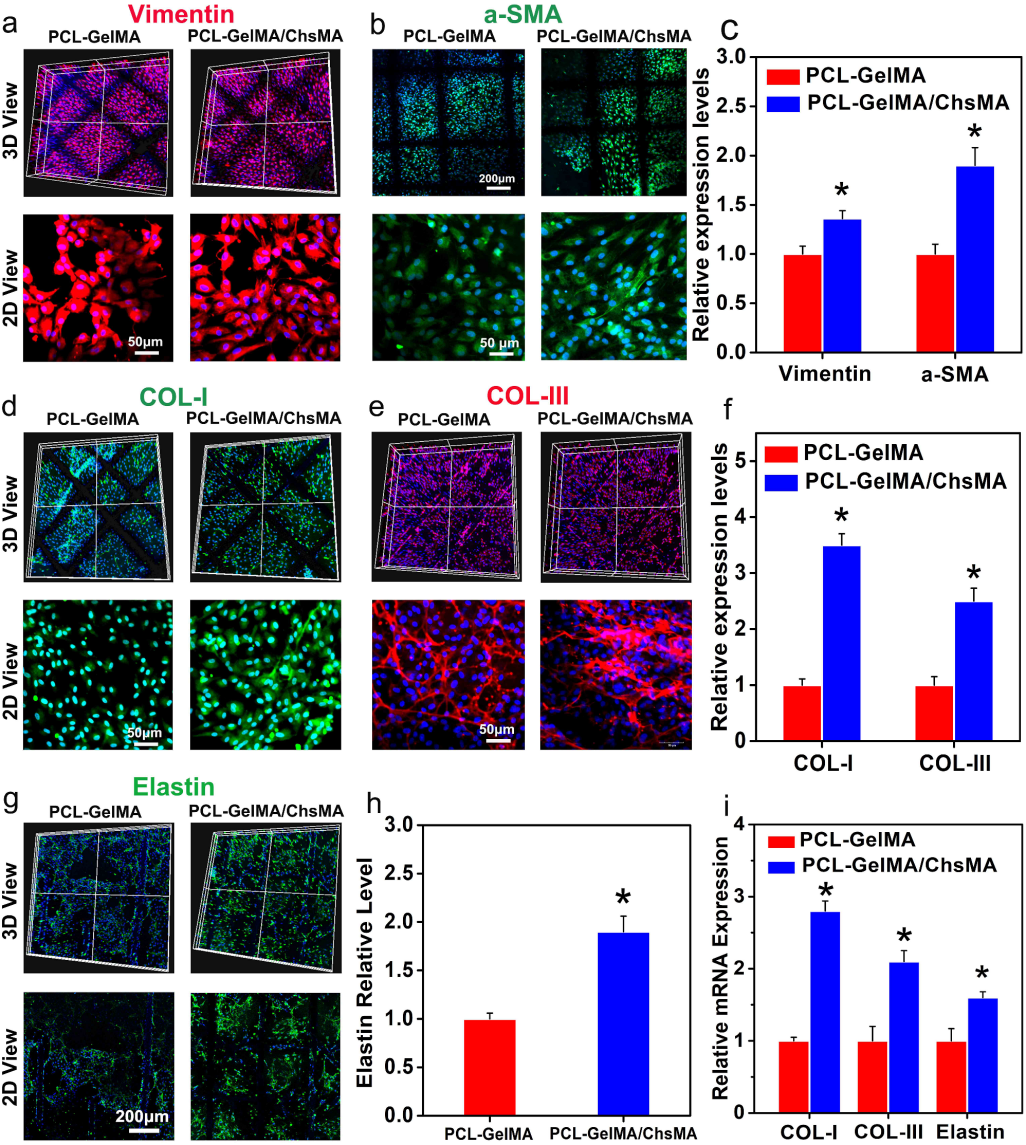
Immunofluorescence analysis of the extracellular matrix remodeling of the 3D-cultured VIC cells. (**a**) Vimentin expression presented by the VIC cells 3D-cultured within PCL-GelMA and PCL-GelMA/ChsMA scaffolds. (**b**) a-SMA expression presented by the VIC cells 3D-cultured within PCL-GelMA and PCL-GelMA/ChsMA scaffolds. (**c**) Quantitative analysis of the immunostaining images in (**a**) and (**b**). (**d**) COL-I expression presented by the VIC cells 3D-cultured within PCL-GelMA and PCL-GelMA/ChsMA scaffolds. (**e**) COL-II expression presented by the VIC cells 3D-cultured within PCL-GelMA and PCL-GelMA/ChsMA scaffolds. (**f**) Quantitative analysis of the immunostaining images of (**d**) and (**e**). (**g**) Elastin expression presented by the VIC cells 3D-cultured within PCL-GelMA and PCL-GelMA/ChsMA scaffolds. (**h**) Quantitative analysis of the immunostaining images in (**g**). (**i**) The mRNA expression level of COL-I, COL-III and Elastin in VICs after culturing within different scaffolds for 14 days. Data represent means ± SD (*n* = 6). *p* < 0.05* indicates a significant difference compared with the PCL-GelMA group

To evaluate the suitability of PCL-GelMA/ChsMA scaffolds for HVTE, GelMA/ChsMA hydrogels laden with primary VICs were incorporated into MEW-PCL scaffold through molding. This method produced hybrid constructs that leverage the customized mechanical characteristics and biomimetic microarchitecture offered by the PCL fiber phase, along with the heightened ECM production commonly observed in cell-laden hydrogels. Previous reports have indicated that healthy adult VICs display a quiescent fibroblast-like phenotype, expressing vimentin and, to a lesser extent, αSMA (a myofibroblast marker) [48, 49]. Nevertheless, valves subjected to irregular hemodynamic circumstances have shown the presence of activated VICs. These activated VICs, identified as myofibroblasts, initiate a proliferation process and actively participate in the extensive remodeling of the ECM. [50] As illustrated in Fig. 6a-c, the VICs encapsulated within all hydrogel samples displayed the expression of both α-SMA and vimentin following a 14-day cultivation period, signifying the transition of VICs from a fibroblastic to a myofibroblastic phenotype.

To further assess the ECM deposition and remodeling capabilities of VICs seeded in the hydrogel-embedded PCL scaffolds, biological assays were conducted to quantify the contents of collagen I, collagen III, and elastin after 14 days of culture. The incorporation of ChsMA facilitated increased deposition of collagen and elastin. As depicted in Fig. 6d-f, VICs within the PCL-GelMA/ChsMA scaffold exhibited significantly higher levels of collagen I and collagen III compared to those within the PCL-GelMA scaffold. Furthermore, VICs in the PCL-GelMA/ChsMA scaffold also demonstrated significantly higher levels of elastin compared to those in the PCL-GelMA scaffold (Fig. 6g, h). The gene expression levels of collagen I, collagen III, and elastin were evaluated after 14 days of VICs culture in scaffolds. VICs cultured in PCL-GelMA/ChsMA demonstrated a heightened gene expression for collagen I, collagen III, and elastin, as illustrated in Fig. 6i. These findings substantiate our hypothesis that the synergistic combination of bioactive GelMA/ChsMA hydrogel with anisotropic PCL MEW scaffolds has a positive impact on cell-scaffold interactions, particularly in terms of ECM secretion and deposition.

The anticalcification ability of engineered scaffolds is an assuredly important property for HVTE scaffold. GelMA/ChsMA hydrogels laden with VICs were incorporated into MEW-PCL scaffold and cultured in a pro-osteogenic environment using osteogenic differentiation medium (ODM) for 14 days to explore how the scaffold components affected the osteogenic differentiation of VICs. ODM supports VICs proliferation, with no significant cytotoxicity observed (Figure S3a). It was found that large areas of Alizarin Red staining (ARS) and quantitative analysis were observed on both the PCL and PCL-GelMA scaffolds, lower ARS was detected on the PCL-GelMA/ChsMA scaffold (Figure S3b, c). To further validate the phenotypic change of VICs seeded in the three different HVTE scaffolds in ODM, IF staining was carried out to visualize the expression of Runx2 (an essential transcription factor for osteogenic differentiation), (Figure S3d, e). The VICs cultured in the PCL-GelMA/ChsMA scaffold exhibited the lowest expression of Runx2. RT-qPCR analysis was utilized to determine the expression of two gene markers, including alkaline phosphatase (ALP) and Runx2. It was found that the VICs cultured in the PCL-GelMA/ChsMA scaffold significantly downregulated the expressions of ALP and Runx2 genes compared to the VICs cultured in the PCL and PCL-GelMA scaffolds (Figure S3f, g). These results demonstrated that the introduction of ChsMA into the HVTE scaffolds effectively reduced VICs calcification.

### In vivo immuno-inflammation and calcification analysis of the scaffolds

**Fig. 7:**
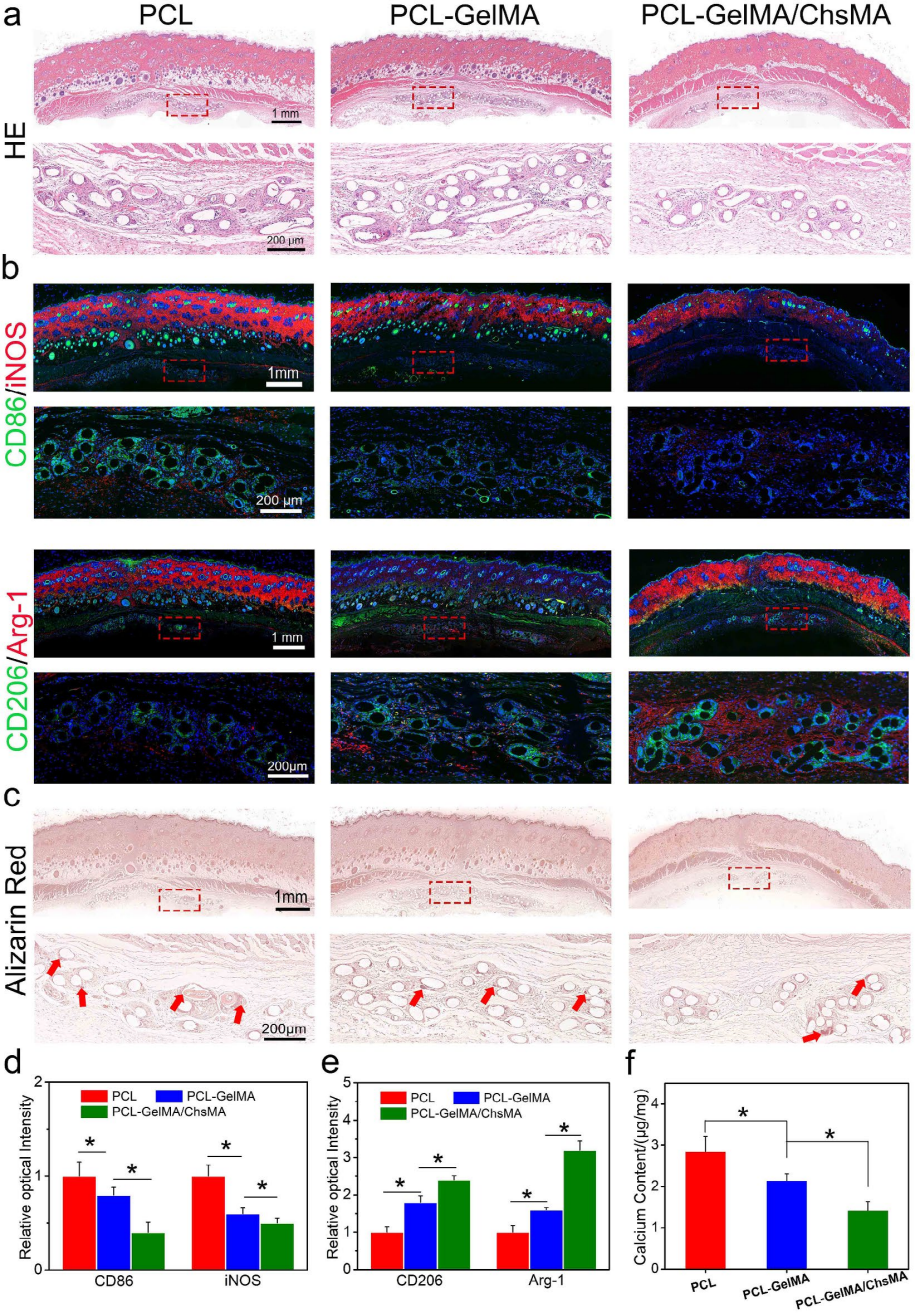
Immune responses induced by PCL, PCL-GelMA and PCL-GelMA/ChsMA scaffolds after subcutaneous implantations for 4 weeks. (**a**) H&E staining of the scaffolds after 4-week implantation. (**b**) Representative immunofluorescence staining images of the scaffolds after 4-week implantation (CD86/iNOS for M1 subtype macrophage, CD206/Arg-1 for M2 subtype macrophage). (**c**) Alizarin red staining showed calcification of the scaffolds after 4-week implantation (red arrows represent calcification). (d-e) Quantitative analysis of the immunostaining images of CD86, iNOS, CD206 and Arg-1. (**f**) Calcium contents of PCL, PCL-GelMA and PCL-GelMA/ChsMA scaffolds after 4-week implantation, and the all samples were measured by ICP. Data represent means ± SD (*n* = 6). *p* < 0.05* indicates a significant difference

The rat subcutaneous implantation model was employed to evaluate cellular infiltration, inflammation, and calcification resistance in vivo. Figure 7a presents a series of hematoxylin and eosin (H&E) staining images depicting PCL, PCL-GelMA, and PCL-GelMA/ChsMA scaffolds. After 30 days of implantation, all three types of scaffolds displayed a dense tissue capsule surrounding the samples. The PCL fibers, being non-dyeable, resulted in a porous representation of fiber cross-sections in the sections. H&E staining aided in distinguishing nuclei, appearing as deep purple, and infiltrated connective tissue, exhibiting pink staining. Remarkably, Fig. 7a observations revealed the absence of a significant inflammatory response or damage in the adjacent tissues, indicating that all PCL scaffolds exhibited good in vivo biocompatibility.

Macrophages are adaptable cells capable of transitioning into various states when exposed to different stimuli. To characterize the polarization of macrophages after a 30-day implantation, triple-labeled immunofluorescence was conducted. M1-polarized macrophages were identified via co-staining of CD86 and iNOS, while M2-polarized macrophages were labeled by CD206 and arginase-1 (Arg-1). Notably, fewer M1-polarized macrophages and an increased presence of M2 macrophages were observed around PCL-GelMA/ChsMA scaffolds compared to the other groups (Fig. 7b-e). These findings suggested a tendency for macrophages around PCL-GelMA/ChsMA scaffolds to polarize towards the M2 phenotype. The favorable anti-inflammatory performance of PCL-GelMA/ChsMA scaffolds might be attributed to the anti-inflammatory properties of chondroitin sulfate, a sulfur-containing polysaccharide known for its certain anti-inflammatory and immunomodulatory capabilities [51, 52]. Additionally, the excellent biocompatibility of the hydrogel coating may contribute to the reduced likelihood of immune rejection.

Sections of the three samples exhibited signs of calcification in both the materials and their surrounding tissues (Fig. 7c). Following a 30-day implantation period, alizarin red staining revealed calcification deposits (dark-red portions) on the PCL scaffold, while no calcification was observed on PCL-GelMA or PCL-GelMA/ChsMA scaffolds. The calcification also appeared to extend into the surrounding tissues, particularly at the interface between the tissue and the PCL scaffold. In the rest of the encapsulated tissue, little to no calcification was present. To validate these histological findings, a calcium content assay was conducted. ICP-OES was utilized for quantitative analysis of calcification. After a 30-day implantation in SD rats, the calcium content of PCL, PCL-GelMA, and PCL-GelMA/ChsMA scaffolds was measured at 2.8, 2.1, and 1.7 µg/mg, respectively (Fig. 7f). This result indicates that PCL-GelMA/ChsMA exhibits potential anti-calcification properties.

### Overall scaffold performance under hemodynamic conditions

**Fig. 8:**
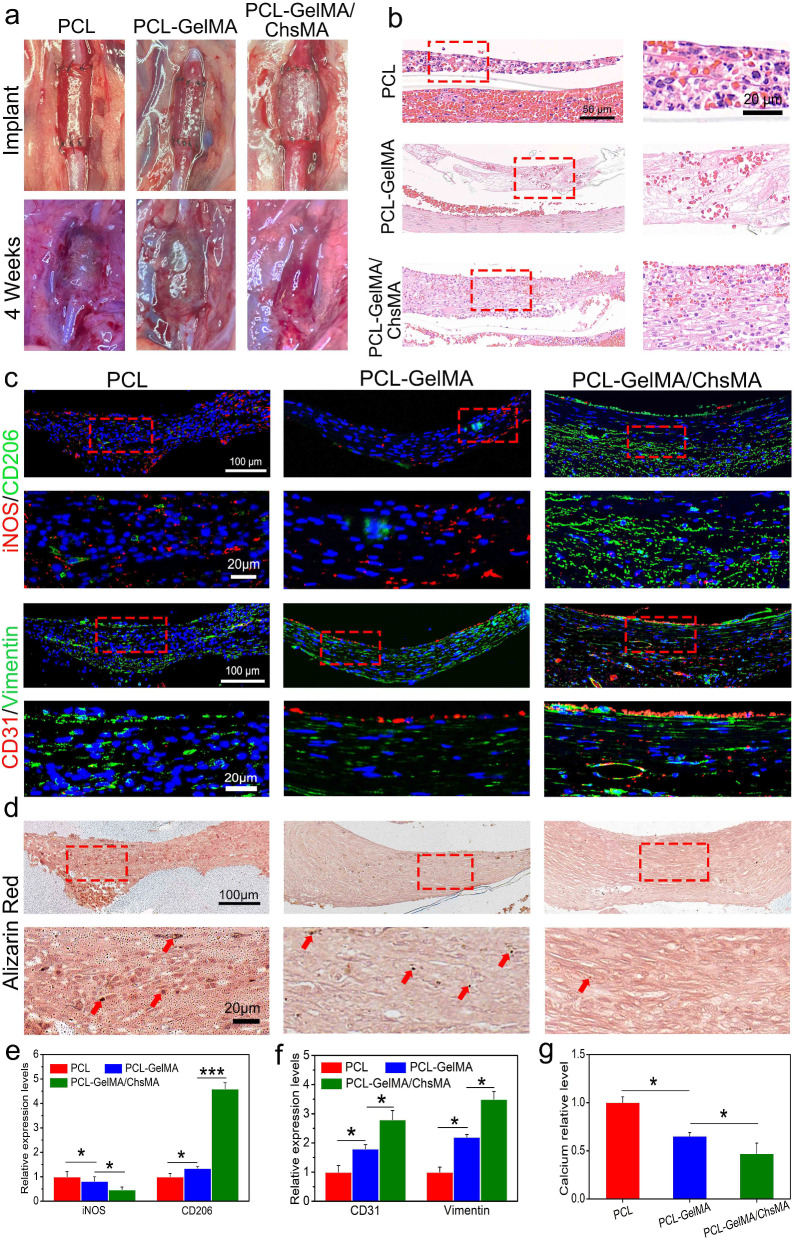
Establishment of the abdominal aorta model and overall assessment of the HVTE scaffolds after a 4-week implantation. (**a**) Photographic representation of the scaffolds implanted in the rat abdominal aorta. (**b**) H&E staining of the scaffolds after 4-week implantation. (**c**) Immunofluorescence images of iNOS and CD206 illustrating inflammatory infiltration, CD31 and vimentin displaying cellularization of the scaffolds. (**d**) Alizarin red staining revealed the occurrence of calcification in the scaffolds (red arrows represent calcification). (e-g) Semi-quantitative analysis of immunostaining images of iNOS, CD206, CD31, Vimentin and calcification. Data represent means ± SD (*n* = 6). *p* < 0.05*, *p* < 0.01*** indicates a significant difference

To evaluate scaffold performance in a hemodynamic environment, we established a rat abdominal aorta implantation model. After 4 weeks of implantation, the PCL scaffold exhibited signs of thrombogenesis (Fig. 8a) and underwent significant dilation, resembling an aneurysm, while the PCL-GelMA and PCL-GelMA/ChsMA scaffolds maintained their tubular structure. Hematoxylin and eosin (H&E) staining images (Fig. 8b) revealed a layer of cell infiltration on the inner surface of the scaffold. Notably, the cells infiltrating the inner surface of the PCL scaffold exhibited a higher presence of inflammatory cells, whereas the PCL-GelMA and PCL-GelMA/ChsMA scaffolds displayed reduced inflammatory cell infiltration. These findings indicate that the PCL-GelMA and PCL-GelMA/ChsMA scaffolds exhibited favorable biocompatibility within the hemodynamic environment.

Post-implantation inflammation of the scaffolds was evaluated using immunofluorescence staining for iNOS (indicative of M1 subtype macrophages) and CD206 (indicative of M2 subtype macrophages) (Fig. 8c, e). Overall, all scaffolds exhibited some degree of inflammation, with PCL scaffolds demonstrating a more pronounced inflammatory response compared to PCL-GelMA and PCL-GelMA/ChsMA scaffolds. Following a 4-week implantation period, CD206 M2 macrophages, indicative of immunoregulation, dominated within the scaffolds, while the presence of iNOS M1 macrophages, associated with immune stimulation, was minimal. This observation suggests a subsiding acute inflammatory response, with ongoing tissue remodeling in the scaffolds at this juncture. In contrast to PCL and PCL-GelMA scaffolds, PCL-GelMA/ChsMA scaffolds displayed an earlier transition from the acute inflammatory phase to tissue repair and remodeling, suggesting their potential anti-inflammatory functionality.

Endothelialization holds paramount importance for TEHV scaffolds. Its importance stems not only from its vital role in integrating and regenerating TEHV to mimic a native valve but also from its diverse physiological functions. These functions encompass the maintenance of blood homeostasis, the formation of an immune barrier, and the regulation of the phenotype of VICs [52]. Immunofluorescence staining for CD31 and vimentin was employed to visualize endothelialization and cellularization (CD31 for endothelial cells and vimentin for interstitial cells). As depicted in Fig. 8c and f, CD31 staining revealed the formation of a confluent CD31 + endothelial cell monolayer in the PCL-GelMA/ChsMA scaffold, albeit with some incomplete sections still present. The inner surface of the PCL-GelMA scaffold exhibited only a sparse presence of endothelial cells, while no endothelialization was observed in the PCL scaffold. Vimentin staining outcomes suggested that both PCL-GelMA and PCL-GelMA/ChsMA scaffolds underwent cellularization, wherein endothelial cells formed a monolayer on the inner surface, and interstitial cells infiltrated into the main body of the scaffolds. This observed pattern resembled the cellular composition and structure of native valves.

Alizarin red stain was employed to assess the calcification of these scaffolds under a hemodynamic environment. After 4-week implantation, alizarin red stained calcification deposits (brown portions) were observed in the PCL scaffold group, and a few calcifications were present in the PCL-GelMA scaffold group (Fig. 8d, g). In contrast, no visible brown portions were detected on PCL-GelMA and PCL-GelMA/ChsMA, signifying the absence of significant calcification after 4-week implantation. As a result, we introduced an innovative approach by suturing the scaffold into a tubular graft, which was subsequently implanted in the abdominal aorta to partially emulate the hemodynamic environment of a native valve. This model demonstrates that the PCL-GelMA/ChsMA scaffold possesses immunomodulatory effects, excellent hemocompatibility, and endothelialization, particularly in terms of recruiting and capturing endothelial cells from peripheral blood sources, coupled with anti-calcification ability.

## Conclusions

In this investigation, we employed melt-electrowriting techniques to fabricate tri-layered biomimetic PCL scaffolds for HVTE. The biomimetic MEW-PCL scaffolds were combined with a microporous bioactive hydrogel (GelMA/ChsMA), providing both the necessary structural integrity to withstand challenging cardiovascular loading conditions and sufficient porosity for facilitating cell infiltration. The composite PCL-GelMA/ChsMA HVTE scaffolds offer significant advantages, exhibiting tunable anisotropic mechanical properties reminiscent of the structural and mechanical characteristics of native heart valves. The incorporation of ChsMA enhances the hemocompatibility and endothelialization of HUVECs in vitro. Moreover, the PCL-GelMA/ChsMA scaffolds supported the growth of VICs within the 3D structure, fostering the remodeling of the heart valve ECM. Upon in vivo implantation in SD rats, the PCL-GelMA/ChsMA scaffolds effectively suppressed immune responses and calcification when compared to the PCL scaffold. These findings suggest that this newly-developed TEHV scaffold addresses the limitations of mechanical and bioprosthetic valves, meeting the clinical demand for prosthetic valves in practical applications.

### Electronic supplementary material

Below is the link to the electronic supplementary material.

Supplementary Material 1

## Data Availability

No datasets were generated or analysed during the current study.
