# Supplementary material for: Melt-electrowriting-enabled anisotropic scaffolds loaded with valve interstitial cells for heart valve tissue Engineering
Source: J Nanobiotechnology. 2024 Jun 28;22:378. doi: 10.1186/s12951-024-02656-5 (PMC11212200; doi:10.1186/s12951-024-02656-5)
Supplement: Supplementary file 1 — Supplementary Material 1 [file 12951_2024_2656_MOESM1_ESM.docx]

**Supporting Information**

**Melt-electrowriting-enabled Anisotropic Scaffolds Loaded with Valve Interstitial Cells for Heart Valve Tissue Engineering**

*Chao Xu^1,#^, Kun Yang^2,#^, Yin Xu^3,#^,* *Xiangfu Meng^4^, Ying Zhou^3^, Yanping Xu^2^, Xueyao Li^2^, Weihua Qiao^3^, Jiawei Shi^3^, Donghui Zhang^4^, Jianglin Wang^2^, Weilin Xu^1^, Hongjun Yang^1,^* , Zhiqiang Luo^2,^*, Nianguo Dong^3,^**

Dr. C. Xu, Prof. W. Xu, Prof. H. Yang

^1^ College of Materials Science and Engineering, State Key Laboratory of New Textile Materials and Advanced Processing Technology, Wuhan Textile University, Wuhan 430200, China

E-mail address: [h_j.yang@yahoo.com](mailto:h_j.yang@yahoo.com) (H. Yang)

K. Yang, Y. Xu, X. Li, Prof. J. Wang, Prof. Z. Luo

^2^ College of Life Science and Technology, Huazhong University of Science and Technology, Wuhan 430074, China

E-mail address: [zhiqiangluo@hust.edu.cn](mailto:zhiqiangluo@hust.edu.cn) (Z. Luo)

Y. Xu, Dr. Y. Zhou, Prof. W. Qiao, Prof. J. Shi, Prof. N. Dong

^3^ Department of Cardiovascular Surgery, Union Hospital, Tongji Medical College, Huazhong University of Science and Technology, Wuhan, 430000 China

E-mail address: dongnianguo@hotmail.com (N. Dong)

X. Meng, Prof. D. Zhang

^4^ State Key Laboratory of Biocatalysis and Enzyme Engineering, School of Life Science, Hubei University, Wuhan 430062, China

^#^The authors equally contribute to this work.

**Supplementary Materials and Methods**

**Isolation and culture of VICs:** VICs were isolated from 2-year-old porcine aortic valve (AV) leaflets. Briefly, the surfaces of the AV leaflets were digested with collagenase Ⅰ (2mg mL^-1^) for 10 min to remove the endothelium. The remaining portions of the leaflets were then cut into small pieces (~ 1 mm × 1 mm) and digested with fresh collagenase Ⅰ (2mg mL^-1^) solution for 6 h. After filtration and centrifugation, the cells were pooled together and seeded in three wells of 6-well plate to generate 3 replicates. The VICs were cultured in Dulbecco’s Modified Eagle’s Medium (DMEM, Invitrogen) supplemented with 10% fetal bovine serum (FBS, Gibco) and 1% penicillin/streptomycin (PS, Gibco) at 37 °C and 5% CO_2_.

**Calcification analysis:** 2 × 10^5^ VICs mixed with hydrogel and was injected into a PCL scaffold, cultured in an osteogenic differentiation medium (ODM) for 14 days. The ODM for VICs induction contained 10 mM β-glycerophosphate, 50 μg/ml L-ascorbic acid, and 100 nM Dexamethasone. The ODM was changed every 3 d until the predetermined time point reached.

To quantify calcified matrix developed in different experimental conditions, an Alizarin Red absorbance assay was implemented. Hydrogels scaffolds were fixed with paraformaldehyde and then incubated with Alizarin Red S (ARS) dye. Bound ARS dye was then released from the gels using 10% acetic acid, followed by neutralization with 10% ammonium hydroxide. The concentration of dye in solution was then quantified using absorbance spectroscopy at 405 nm wavelength.

The scaffolds samples were incubated overnight at 4°C with primary antibodies specific to Runx2 (ab76956, abcam). Following the primary antibody incubation, the samples were exposed to secondary fluorescent antibodies for 2 hours and subsequently treated with DAPI nuclear dye for 10 minutes. Finally, the stained samples were visualized using a Confocal Laser Scanning Microscope (FV3000, Olympus).

**Rat subdermal implantation model establishment:** A rat subdermal implantation model was used to evaluate the histocompatibility of the scaffolds. In brief, scaffolds were cut in half, and one half was used for implantation and the other one as pre-implant control. Eighteen SD rats (male, 190-210 g) were randomly divided into three groups and anesthetized with continuous inhalation of 4% isoflurane. Small midline abdominal incisions were made and the scaffolds were implanted into the subcutaneous pockets formed by blunt dissection. The incisions were sutured with 5-0 prolene suture. After 4 weeks, animals were sacrificed and the scaffolds were removed.

**Rat abdominal aorta implantation model establishment:** Specifically, scaffolds were trimmed into the rectangular piece with dimension of 5 mm × 4 mm, and then rolled up along the long axis and sewn as the tubular scaffolds. Thirty SD rats (male, 190-210 g) were randomly divided into three groups and anesthetized with isoflurane (4%), and the abdominal cavity was cut open along the mid ventral line and the abdominal aorta was exposed with protective removal of the intestinal tube. After carefully dissociating, the abdominal aorta was temporarily occluded with artery clip and cut open, and the broken ends were immediately flushed with heparinized saline solution. The tubular scaffolds were end-to-end anastomosed into the abdominal aorta with 8-0 prolene sutures. Then the artery clip was removed and the blood flow was recovered with gently compress to stop bleeding. Intestines were returned and the incision was closed with interrupted 5-0 sutures. Rats were successfully awakened from anesthesia, and the survival and the lower limbs mobility were monitored every day.

**RT-qPCR assay:** Total RNA was extracted from the cells cultured in the different samples by TRIzol (Invitrogen, Carlsbad, CA, USA) 14 days of culture. The cDNA was generated using oligo-dT primers (oligo-dT primers (Promega, USA)) according to the manufacturer's protocol. Quantitative PCR was performed with SYBR Green Premix Ex Taq (Takara, Kyoto, Japan) and then detected by using an RT-PCR system (Takara, Kyoto, Japan). The expression of target genes (HIF-1α, VEGF, vWF, bFGF in HUVEVs and COL-Ⅰ, COL-Ⅲ, Elastin in VICs) were normalized against β-actin. Relative gene expression values were calculated by 2^-ΔΔCt^ method. The primers for RT-qPCR were described in Table S1.

**Supplementary figures**


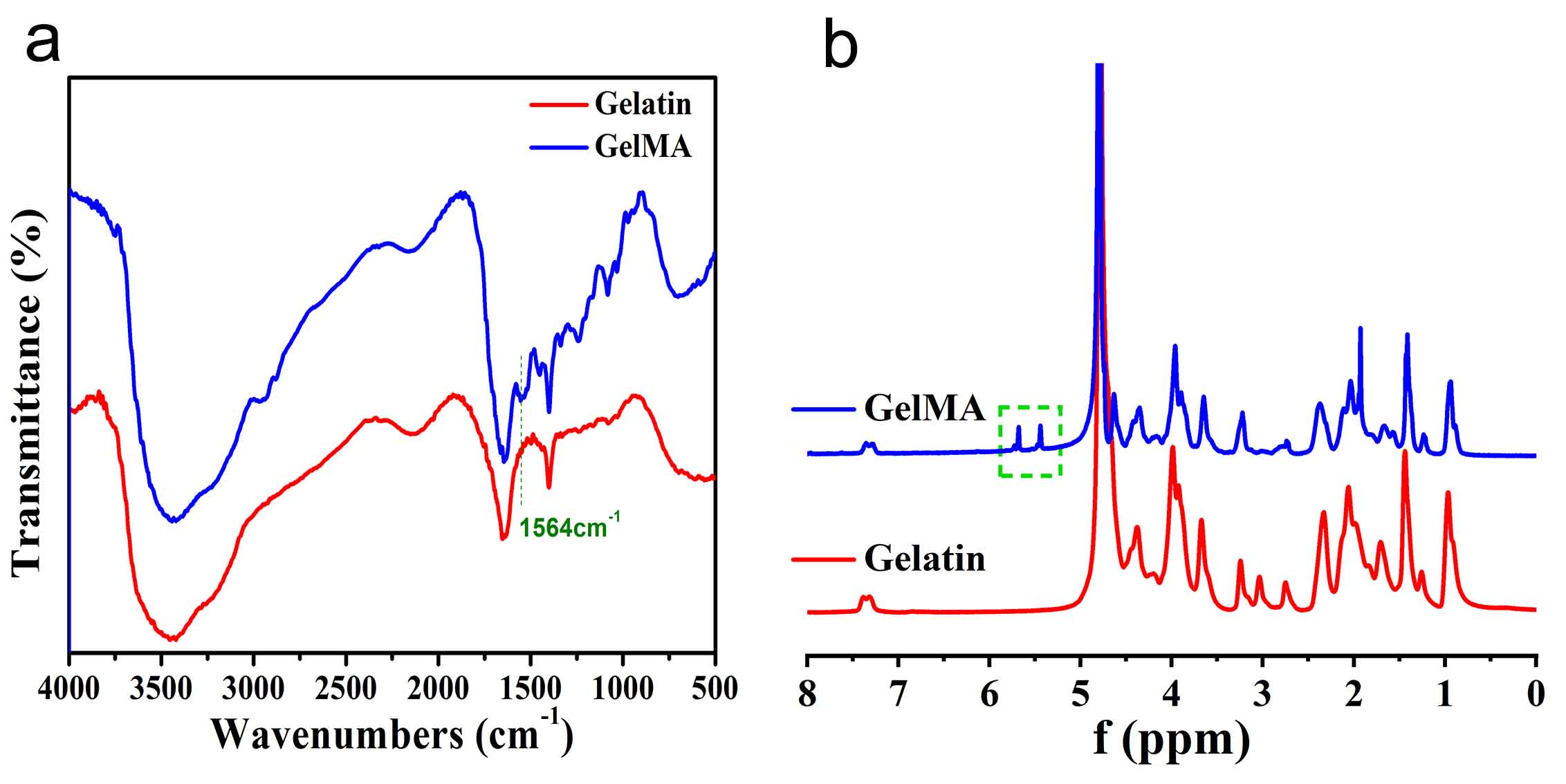


**Figure S1**. Characterization of GelMA. (b) FTIR spectra of before and after modification of gelatin with methacrylate anhydride. (c) ^1^H NMR spectra of gelatin and GelMA.


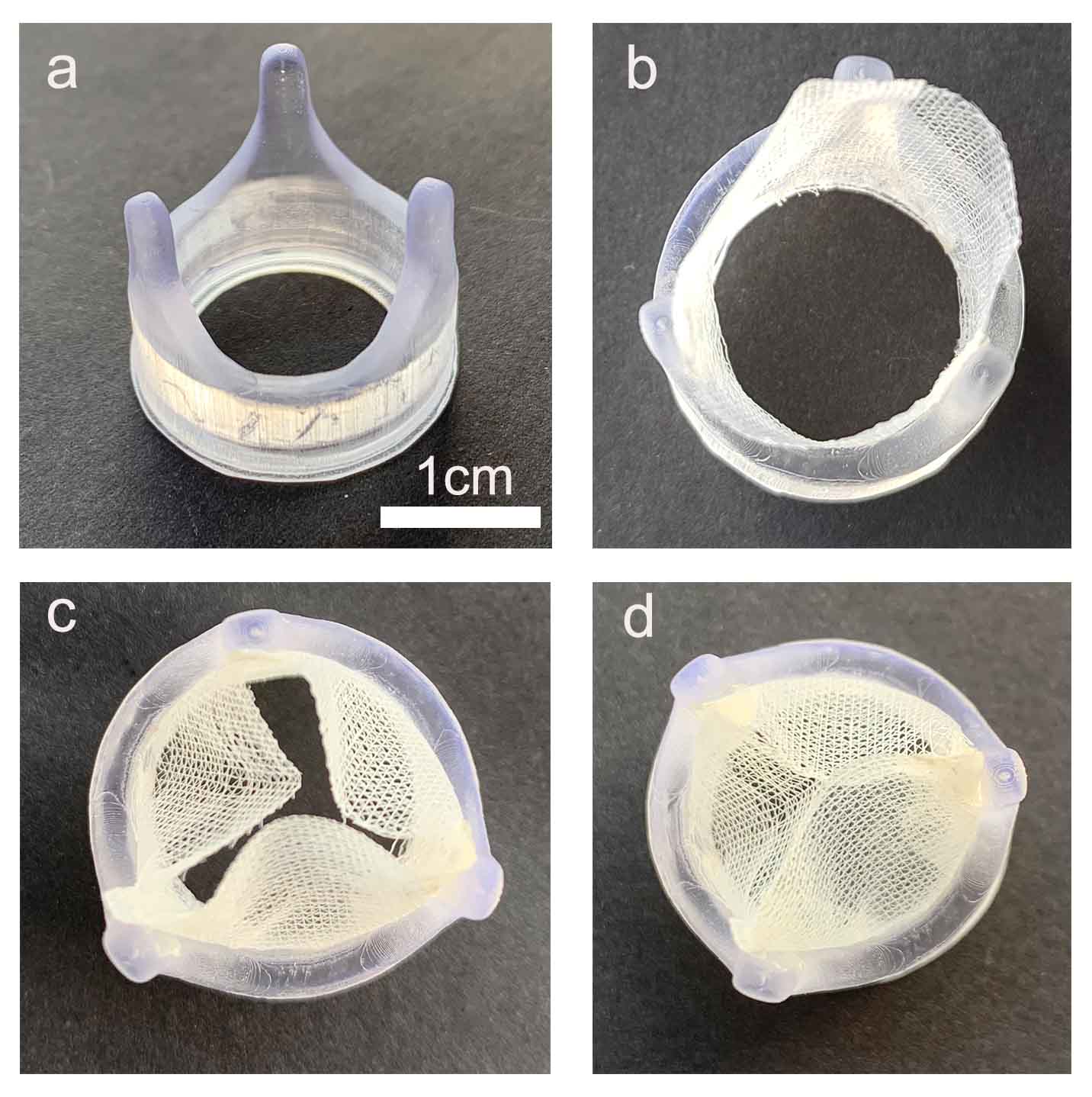


**Figure S2**. TEHV PCL-GelMA/ChsMA prototype components. (a) Valve stent of 20 diameter was designed using SolidWorks. PCL-GelMA/ChsMA leaflet via cyanoacrylate fixed to the Valve stent assembled TEHV, in the (b) open, (c) semi-closed and (d) tightly closed state.


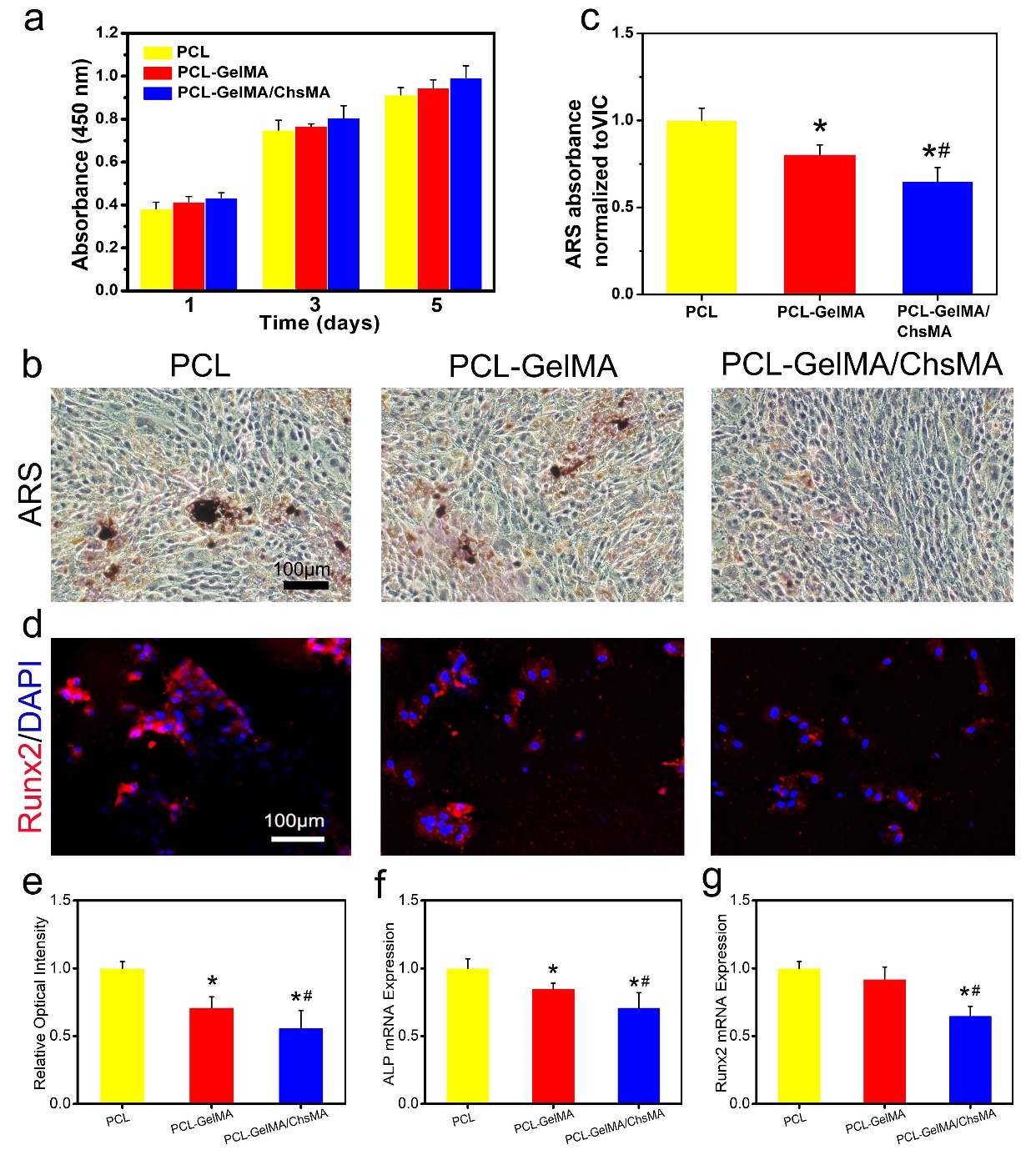


**Figure S3**. Calcification analysis of VICs in osteogenic differentiation medium. (a) Cell viability of VICs cultured with PCL, PCL-GelMA and PCL-GelMA/ChsMA scaffolds. (b) Representative images Alizarin Red S staining of calcium deposition. (c) Quantitative analysis of Alizarin Red S concentration following dye extraction from stained scaffolds. (d) Immunofluorescent staining of Runx2 (red) and nuclei (blue) of VICs seeded in PCL, PCL-GelMA and PCL-GelMA/ChsMA scaffolds and cultured in ODM. (e) Quantitative analysis of the immunostaining images of Runx2. (f, g) mRNA expression on day of VICs cultured with the three different scaffolds and cultured in ODM. (p < 0.05* indicates a significant difference compared with the PCL group, p < 0.05# indicates a significant difference compared with the PCL-GelMA group.)

**Supplementary Table**

**Table 1** The primer sequences for each primer used in the qRT-PCR.

| **Genes** | **Primer sequences** | **Annealin temperature (^o^C)** |
| --- | --- | --- |
| **Pig species** | | |
| COL-Ⅰ | Forward: TGAAGCTGGTCCCCAAGGA | 60 |
|  | Reverse: AGCACCAGCAATACCAGGAG |  |
| COL-Ⅲ | Forward: CTAGCCGAGCTTCCCAGAAC | 60 |
|  | Reverse: CCCCATTCCCCAGTGTGTTT |  |
| Elastin | Forward: ACCCTCCTCGAGGTTACAGC | 60 |
|  | Reverse: TGACGAAGGTCTCCACTCCA |  |
| **Human species** | | |
| HIF-1α | Forward: TGGTGCTAACAGATGATGGTG | 60 |
|  | Reverse: CATGGTCACATGGATGGGTA |  |
| VEGF | Forward: CACTGGACCCTGGCTTTACT | 60 |
|  | Reverse: GACGTCCATGAACTTCACCA |  |
| vWF | Forward: ATGATTCCTGCCAGATTTGC | 60 |
|  | Reverse: AGACTCTTTGGTCCCCCTGT |  |
| bFGF | Forward: CAATTCCCATGTGCTGTGAC | 60 |
|  | Reverse: ACCTTGACCTCTCAGCCTCA |  |
